# Draft Genome Sequences of Six Flavobacterium psychrophilum Strains Isolated from Dead Juvenile Ayu (Plecoglossus altivelis altivelis) near the Mouth of the Nahari River, Kochi, Japan

**DOI:** 10.1128/MRA.00759-19

**Published:** 2019-07-18

**Authors:** Hazuki Yamashita, Takayuki Wada, Yusuke Kato, Takuji Ikeda, Masayuki Imajoh

**Affiliations:** a Graduate School of Integrated Arts and Science, Kochi University, Nankoku, Kochi, Japan; b Department of International Health, Institute of Tropical Medicine, Nagasaki University, Nagasaki, Japan; c School of Tropical Medicine and Global Health, Nagasaki University, Nagasaki, Japan; d Laboratory of Fish Disease, Aquaculture Course, Department of Marine Resource Science, Faculty of Agriculture and Marine Science, Kochi University, Nankoku, Kochi, Japan; University of Maryland School of Medicine

## Abstract

Flavobacterium psychrophilum causes bacterial cold-water disease in ayu inhabiting clear rivers in Japan. In this study, we report the draft genome sequences of six F. psychrophilum strains isolated from the gills and skin of dead juvenile ayu near the mouth of the Nahari River.

## ANNOUNCEMENT

Ayu (Plecoglossus altivelis altivelis) is a popular angling fish in Japan. During 2006 to 2012, the ayu population in the Nahari River, located in the eastern Kochi Prefecture on Shikoku Island, was estimated to be 145,000 to 1,540,000 individuals, and a decrease of 30% to 76% was observed in the population between May and October of each year (1). One of the possible causes for this decrease in the ayu population is the occurrence of bacterial cold-water disease (BCWD) (1). Flavobacterium psychrophilum, belonging to the family *Flavobacteriaceae,* (2) order *Flavobacteriales* (3), and class *Flavobacteriia* (4), is the etiological agent of BCWD.

In April 2018, there was a mass die-off of juvenile ayu near the mouth of the Nahari River during the beginning of upstream migration season due to the sudden spread of BCWD. Six dead individuals were collected at that time. Five samples from gills and one sample from a skin ulcer were streaked onto each modified cytophaga (MCYT) agar plate (5), followed by incubation at 18°C for 7 days. A single colony was picked from each plate and genetically identified as F. psychrophilum according to the guidelines of the council for the control of BCWD of ayu (http://www.maff.go.jp/j/syouan/suisan/suisan_yobo/ayu_reisui/pdf/sisin_2008.pdf). Herein, we report the draft genome sequences of the isolated strains NNS1-1804, NNS2-1804, NNS4-1804, NNS6-1804, and NNS7-1804 from the gill samples and strain NNS5-1804 from the skin ulcer sample.

Liquid cultures of single colonies of six strains were produced in 10 ml of MCYT broth for 4 days at 18°C. Genomic DNA was extracted using Genomic-tip 500/G columns and buffers (Qiagen) according to the manufacturer’s instructions. Library construction was performed according to the Illumina TruSeq Nano DNA library prep kit protocol and then sequenced on the Illumina MiSeq platform using the MiSeq reagent kit v3 (600 cycles). Raw reads were imported into CLC Genomics Workbench 9.5.3 (Qiagen) with default parameters for trimming, removal of low-quality base calls (quality limit, 0.05; minimum read length, 50 bp), and *de novo* assembly of high-quality reads. Annotation of the assembled data was performed using the DDBJ Fast Annotation and Submission Tool v. 1.1.0, with default settings (6). Table 1 shows the sequencing metrics and genomic data of the six strains. Multilocus sequence typing (MLST) analysis of the six strains was conducted based on DNA sequence variation within the housekeeping genes *gyrB*, *atpA*, *dnaK*, *trpB*, *fumC*, *murG*, and *tuf*, using the Flavobacterium psychrophilum MLST databases (https://pubmlst.org/fpsychrophilum/). By the analysis, strains NNS1-1804, NNS2-1804, NNS5-1804, NNS6-1804, and NNS7-1804 were classified as MLST sequence type 52 (ST52), whereas strain NNS4-1804 was classified as ST45, suggesting the possibility that ST52 caused mortality of ayu during the mass die-off event. It is reported that ST52 is the most frequent sequence type among ayu isolates and is observed in all seasons in Japan (7).

**TABLE 1:** Sequencing metrics and genomic features of six F. psychrophilum strains

Strain	No. of raw reads	Avg length of raw reads (bp)	No. of high-quality reads	Avg length of high-quality reads (bp)	G+C content (%)	No. of contigs	Genome size (bp)	No. of coding sequences	No. of tRNAs	No. of rRNAs	GenBank accession no.	SRA accession no.
NNS1-1804	2,217,200	300.6	1,997,113	231.6	32.4	138	2,715,238	2,380	39	4	BJSV00000000	DRR182481
NNS2-1804	1,857,890	300.6	1,684,576	235.1	32.4	147	2,717,076	2,384	39	3	BJSW00000000	DRR182482
NNS4-1804	1,801,130	300.6	1,641,514	235.4	32.5	153	2,668,540	2,331	38	2	BJSX00000000	DRR182483
NNS5-1804	3,758,832	300.6	3,424,590	235.0	32.4	176	2,759,586	2,414	39	3	BJSY00000000	DRR182484
NNS6-1804	2,026,464	300.6	1,869,939	238.1	32.4	146	2,716,803	2,385	39	1	BJSZ00000000	DRR182485
NNS7-1804	1,754,606	300.6	1,588,862	235.0	32.4	144	2,717,677	2,383	39	3	BJTA00000000	DRR182486

### Data availability.

This whole-genome shotgun project and raw sequencing reads have been deposited in DDBJ/GenBank and the DDBJ/Sequence Read Archive, respectively. The respective accession numbers are listed in Table 1.
